# Recent advances in direct C–H arylation: Methodology, selectivity and mechanism in oxazole series

**DOI:** 10.3762/bjoc.7.187

**Published:** 2011-11-29

**Authors:** Cécile Verrier, Pierrik Lassalas, Laure Théveau, Guy Quéguiner, François Trécourt, Francis Marsais, Christophe Hoarau

**Affiliations:** 1 UMR COBRA 6014 – Université et INSA de Rouen, IRCOF, Laboratoire de Chimie Organique Fine et Hétérocyclique, BP08, 76131 Mont Saint Aignan, France

**Keywords:** ate complex, catalytic direct arylation, mechanism, oxazole, selectivity, transition-metal catalysis

## Abstract

Catalytic direct (hetero)arylation of (hetero)arenes is an attractive alternative to traditional Kumada, Stille, Negishi and Suzuki–Miyaura cross-coupling reactions, notably as it avoids the prior preparation and isolation of (hetero)arylmetals. Developments of this methodology in the oxazole series are reviewed in this article. Methodologies, selectivity, mechanism and future aspects are presented.

## Introduction

**Scheme 1:**
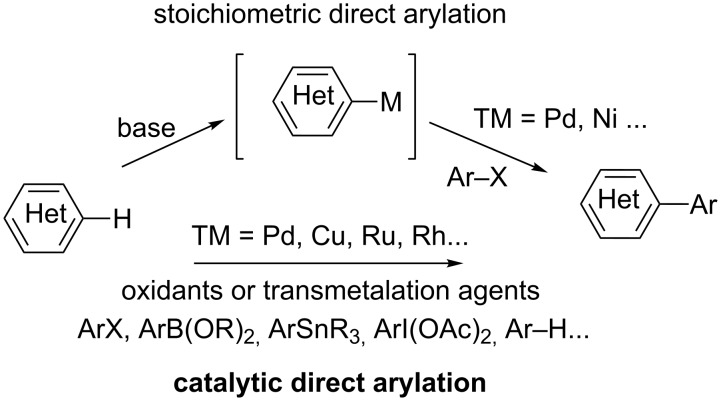
Stoichiometric and catalytic direct (hetero)arylation of arenes.

Deprotonative metalation of aromatics is widely used as a powerful method for regioselective functionalization. Ortho-lithiation by means of alkyllithium and lithium amides bases has been extensively developed as lithiated species display a high reactivity towards many electrophiles, leading to various substitutions (e.g., halogenation, carboxylation, acylation, hydroxymethylation, aminomethylation, sulfuration, oxygenation). However, aryllithiums can rarely be directly involved in transition-metal-catalyzed cross-coupling reactions and are usually transformed into organometallic fragments suitable for efficient Negishi, Stille, Suzuki–Miyaura, and Hiyama cross-coupling reactions [1–2]. Over the past decade, alternatives for more expeditive, practical and chemoselective arylating technics have arisen, thanks mainly to the great development of novel, stoichiometric and catalytic, direct arylation methodologies (Scheme 1).

Thus novel classes of metallating agents resulting from various combinations of organometallic compounds with alkali, including various ate complexes, have been designed, such as zincates (R_2_Zn(TMP)Li·TMEDA) [3–4] ((TMP)_2_Zn·2MgCl_2_·2LiCl) [5], magnesates ((TMP)_3_MgLi, Bu_2_(TMP)MgLi, Bu(TMP)_2_MgLi, (TMP)_4_MgLi_2_) [3,6], aluminates (iBu_3_Al(TMP)Li, Al(TMP)_3_·3LiCl) [3,7], manganate ((Me_3_SiCH_2_)_2_Mn(TMP)Li·TMEDA) [3,8], cuprates (MeCu(TMP)(CN)Li_2_, (TMP)_2_CuLi) [9–10] and cadmium amides ((TMP)_3_CdLi) [11–12], for regio- and/or chemoselective deprotonative metalation of aromatics, producing arylmetal intermediates under smooth reaction conditions that are directly suitable for electrophilic reactions as well as transition-metal-catalyzed cross-coupling reactions. By contrast, the methodology for transition-metal-catalyzed direct arylation [13–18] is based upon the use of various catalytic metalation processes, such as electrophilic metalation, oxidative addition, halogen- or base-assisted metalation–deprotonation, and carbometalation [19–22] combined with diverse functionalizing agents, such as alkenes and alkynes [23], oxidants [24], nucleophiles, organometallics and arenes [25–26] (Scheme 1). In this review we focus on recent developments in catalytic, direct (hetero)arylation of (benz)oxazoles for the preparation of (hetero)aryl(benz)oxazoles, which are common structural units of numerous natural products and are also employed in pharmaceuticals and materials [27].

## Review

### Stoichiometric direct (hetero)arylation of (benz)oxazoles

**Scheme 2:**
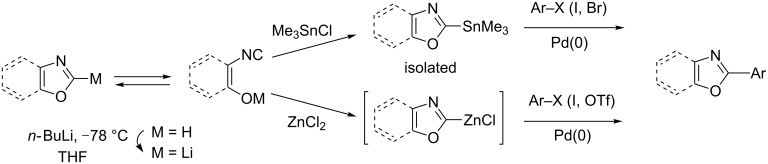
Stille and Negishi cross-coupling methodologies in oxazole series [28,30–31,33–34].

Dondoni first explored the reactivity of the 2-lithio-oxazoles resulting from the ready deprotonation, with *n-*BuLi at low temperature, of the most acidic C2-proton (p*K*_a_ = 20–22 was suggested), which is complicated by the coexistence of a ring-open isonitrile tautomer. In particular, the treatment by trimethylstannylchloride favours the formation of the 2-stannyloxazole, which is isolated as the major product and then successfully engaged in Stille cross-coupling reactions with various (hetero)aryliodides and bromides [28–30]. The subsequent transmetalation reaction following lithiation with zinc dichloride also favours the ring-close oxazole, a trend that is evidenced by ^1^H NMR spectroscopy and attributed to the strong covalent carbon–zinc bond along with the zinc’s low oxophilicity, and this thus allows subsequent palladium-catalyzed Negishi cross-coupling [31–33]. This first, highly efficient, stoichiometric direct arylation of oxazole was further improved for scale-up (Scheme 2) [34].

**Scheme 3:**
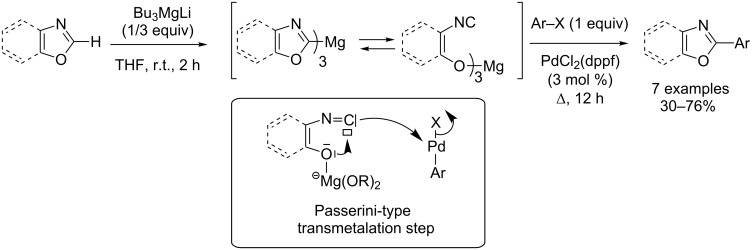
Stoichiometric direct (hetero)arylation of (benz)oxazole with magnesate bases [35].

The C2-magnesation of oxazole was first performed with lithium tributylmagnesate at room temperature, and rapid evolution of the C2-magnesated oxazole to a ring-open isonitrile tautomer was evidenced by ^1^H NMR spectroscopy analysis [35]. Nevertheless, subsequent cross-coupling reactions under palladium catalysis were successfully achieved. Similarly to the Passerini reaction, it was hypothesized that the crucial transmetalation step proceeds throught a nucleophilic displacement of the halogen from the σ-arylpalladium complex by the isonitrile function, leading to the ring-close aryloxazol-2-yl palladium complex delivering products after a final reductive elimination step (Scheme 3).

### Catalytic direct (hetero)arylation of (benz)oxazoles

#### Palladium- and/or copper-catalyzed direct (hetero)arylation with halides: Synthetic methodology

**Scheme 4:**
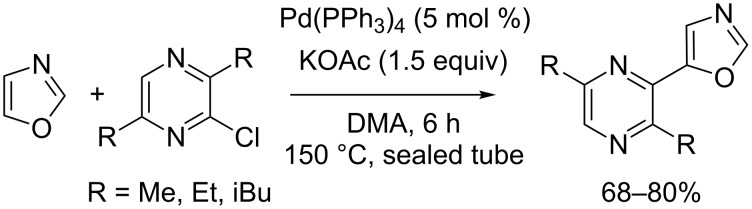
Ohta's pioneering catalytic direct C5-selective pyrazinylation of oxazole [36–37].

The first examples of direct C–H heteroarylation of various azoles were reported by Ohta, including the direct C5-selective pyrazinylation of oxazole with chloropyrazines in the presence of Pd(PPh_3_)_4_ as catalyst and potassium acetate base (Scheme 4) [36–37].

**Scheme 5:**
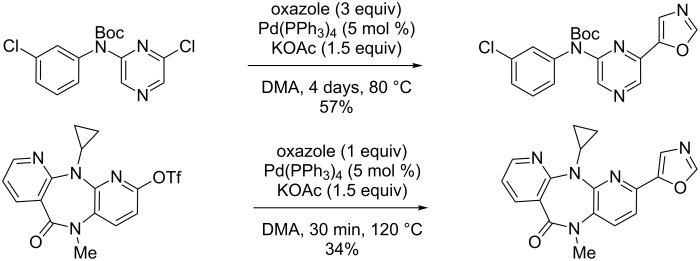
Preparation of pharmaceutical compounds by following the pioneering Ohta protocol [38–39].

**Scheme 6:**
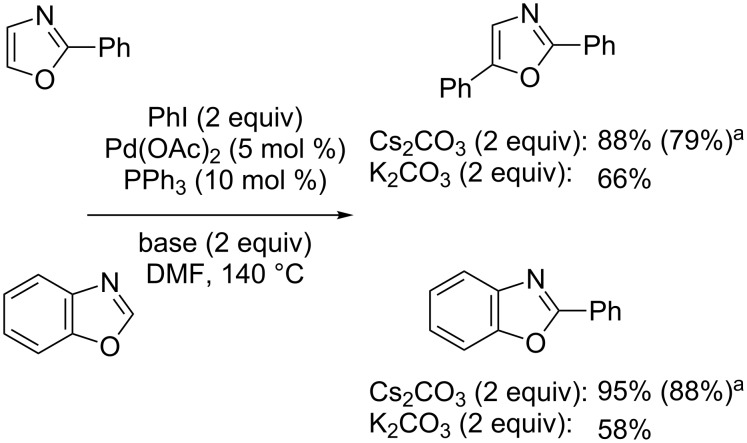
Miura’s pioneering catalytic direct arylations of (benz)oxazoles [40]. ^a^Isolated yield.

**Scheme 7:**
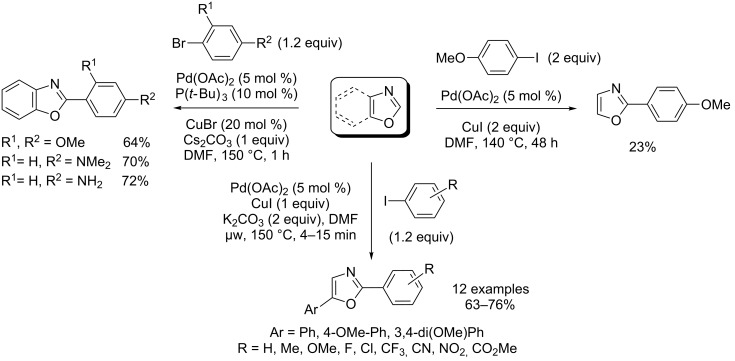
Pd(0)- and Cu(I)-catalyzed direct C2-selective arylation of (benz)oxazoles [41–44].

This protocol was successfully applied to the preparation of two potential inhibitors of vascular endothelial growth factor receptor-2 (Scheme 5) [38–39]. In 1998, Miura reported the first study of Pd(0)-catalyzed direct arylation of imidazoles, oxazoles and thiazoles with iodo- and bromobenzene [40]. It was notably shown that the use of the strong caesium carbonate base led to better results, which was attributed to a better solubility of the base along with a lower solubility of the generated CsI compared to KI salts, preventing an iodide-inhibition effect (Scheme 6). Moreover, copper iodide used as a cocatalyst was able to improve the reactivity at the C2 position significantly. Tamagnan further reported the first example of Pd(0)/Cu(I)-catalyzed direct arylation of benzoxazole (Scheme 7) [41].

Interestingly, Bellina and Rossi reported the Pd(0)/Cu(I)-catalyzed direct arylation of indoles, imidazoles, oxazoles and thiazoles with aryliodides under base-free and ligandless conditions in DMF as solvent [42–43]. However under the developed conditions, the direct coupling of oxazole proved to be inefficient (Scheme 7). More recently, Piguel disclosed an original, ligandless, microwave-assisted, Pd(0)/Cu(I)-catalyzed protocol, which was highly effective in the direct arylation of oxazoles with various arylbromides (Scheme 7) [44].

**Scheme 8:**
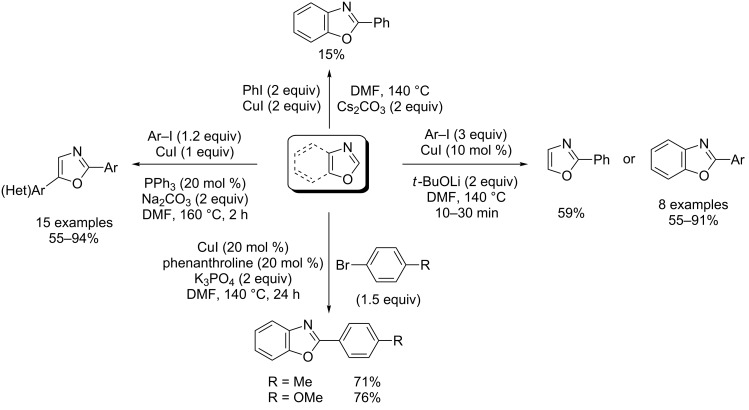
Cu(I)-catalyzed direct C2-selective arylations of (benz)oxazoles [40,45–47].

In his initial study, Miura observed a substantial amount of C2-arylation of azoles, including benzoxazole, using Cu(I) alone as catalyst (Scheme 8) [40]. In 2007, this methodology was judiciously extended by Daugulis who reported a first general methodology for the Cu(I)-catalyzed direct arylation of heterocycles by using aryl iodides electrophiles and based upon the use of lithium *tert*-butoxide as a strong base [45]. In particular, monoarylation of oxazole occurred selectively at the C2 position in 59% yield (Scheme 8). Miura subsequently disclosed the Cu(I)-catalyzed direct arylation of 5-arylated oxazoles with aryl iodides by employing triphenylphosphine ligand and sodium carbonate base (Scheme 8) [46]. Recently, You et al. reported convenient conditions for Cu(I)-catalyzed direct arylation of heterocycles, including electron-rich azoles with aryl bromides, by using potassium phosphate as a base and phenanthroline as a ligand (Scheme 8) [47].

**Scheme 9:**
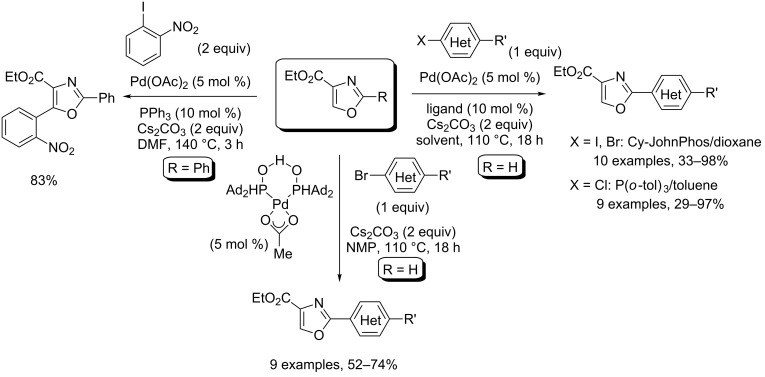
Copper-free Pd(0)-catalyzed direct C5- and C2-selective arylation of oxazole-4-carboxylate esters [48–50,52].

Over the past few decades, it has also been demonstrated that copper catalysis is not required in order to attain good yield and selectivity in Pd(0)-catalyzed direct C–H coupling of azoles, with the main advantage being the possibility to use of a number of Pd salt–ligand combinations to ensure an oxidative addition step and selectivity. Thus, following Miura's catalysis protocol, Hodgetts used the initial ligand to achieve direct C5-arylation of ethyl 2-phenyl-oxazole-4-carboxylate (Scheme 9) [48]. Hoarau selected Cy-JohnPhos and P(*o*-tol)_3_ electron-rich ligands for the direct, C2-selective arylation of ethyl oxazole-4-carboxylate with iodides, bromides and chlorides (Scheme 9) [49–50]. Greaney and Ackermann further revealed the high performance of the Herrmann–Beller precatalyst (HBP) as well as the (1-Ad)_2_P(O)H/Pd(OAc)_2_ combination for the direct coupling of ethyl oxazole-4-carboxylate, with iodides and bromides, respectively (Scheme 9) [51–52].

**Scheme 10:**
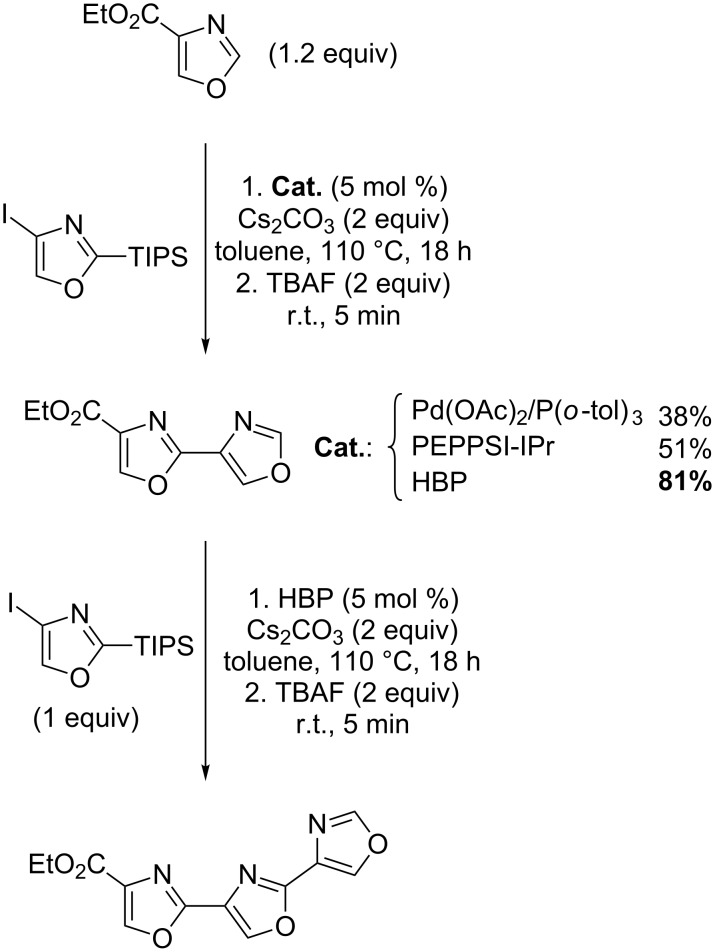
Iterative synthesis of bis- and trioxazoles [51].

Greaney’s methodology was remarkably applied to the preparation of bis- and trisoxazoles units that occur in natural products (Scheme 10).

**Scheme 11:**
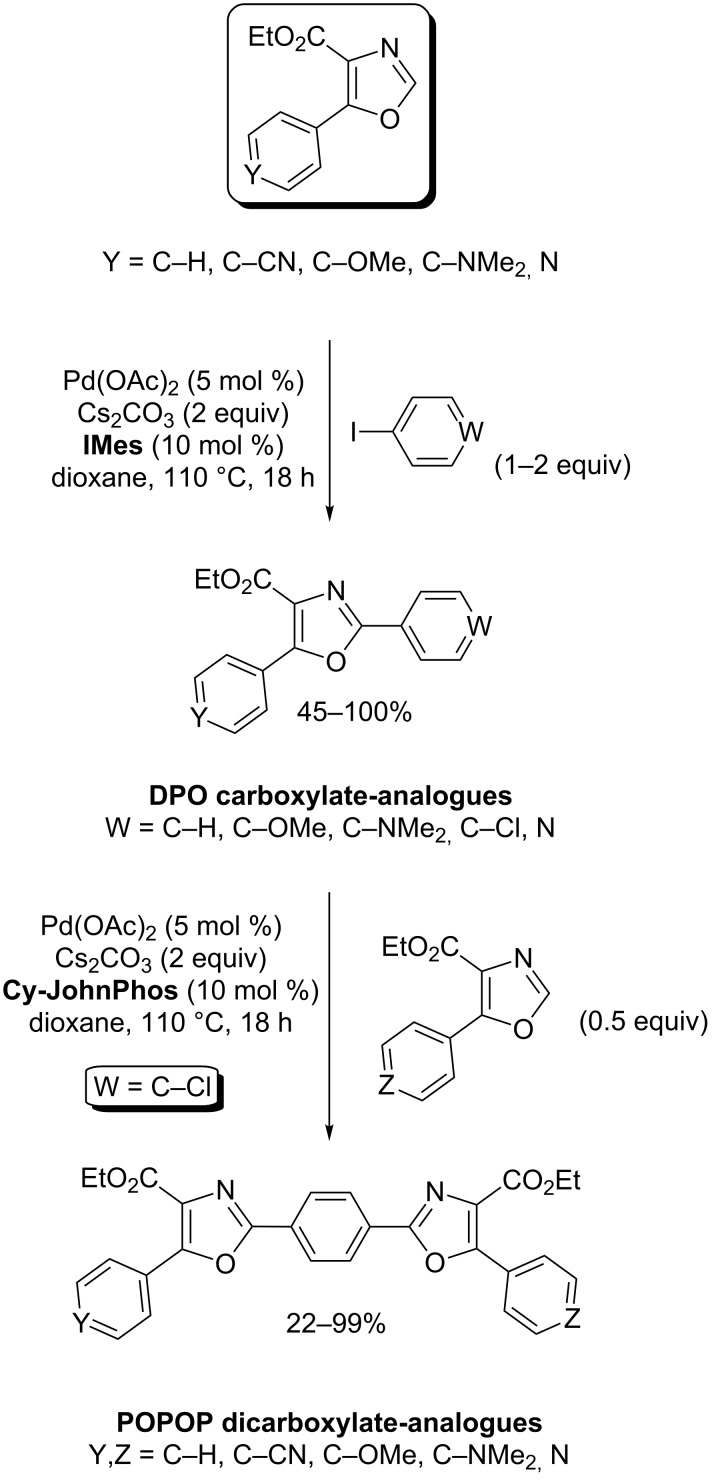
Preparation of DPO- and POPOP-analogues [53].

As an example of application in materials, Hoarau recently reported a novel, sequential, palladium-catalyzed, direct arylation of ethyl oxazole-4-carboxylate, giving a rapid access to DPO and POPOP (di)carboxylate analogues (Scheme 11). Two novel sensors were identified with a two- and three-fold Stokes shift as compared to their DPO (diphenyloxazole) and POPOP (phenyloxazolephenyloxazolephenyl) references, and with high quantum yields (Scheme 11) [53].

**Scheme 12:**
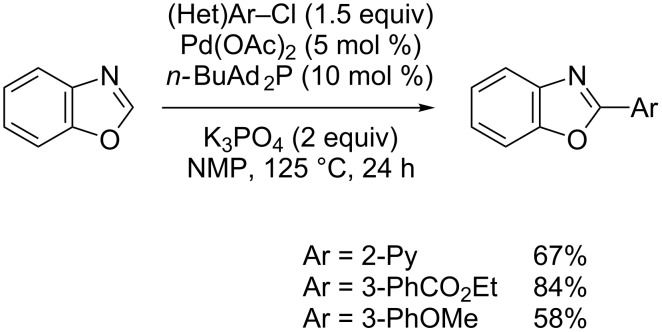
Pd(0)-catalyzed direct arylation of benzoxazole with aryl chlorides [54].

Daugulis used the bulky butyldi-1-adamantylphosphine associated with the potassium phosphate base to achieve Pd(0)-catalyzed direct arylation of various electron-rich heterocycles, including benzoxazole with aryl chlorides (Scheme 12) [54].

**Scheme 13:**
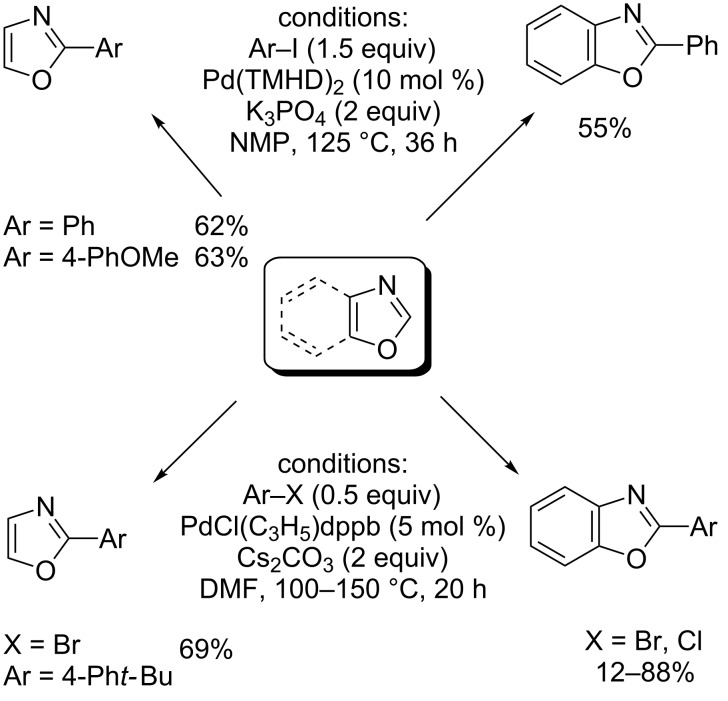
Pd(0)-catalyzed direct C2-selective arylation of (benz)oxazoles with bromides and chlorides using bidentate ligands [55–56].

Bhanage proposed the use of 2,2,6,6-tetramethyl-3,5-heptanedione ligand (TMHD) to achieve regioselective, Pd(0)-catalyzed, direct arylation of *N*-methylindole, thiazoles and oxazoles, using phosphate or carbonate bases (Scheme 13) [55]. The same year, Doucet demonstrated the high efficiency of PdCl(dppb)(C_3_H_5_) precatalyst in Pd(0)-catalyzed direct arylation of thiazoles and oxazoles with arylbromides (Scheme 13) [56]. Notably under these two protocols, oxazole was monoarylated selectively at the C2 position in 62% and 69% yields, respectively.

**Scheme 14:**
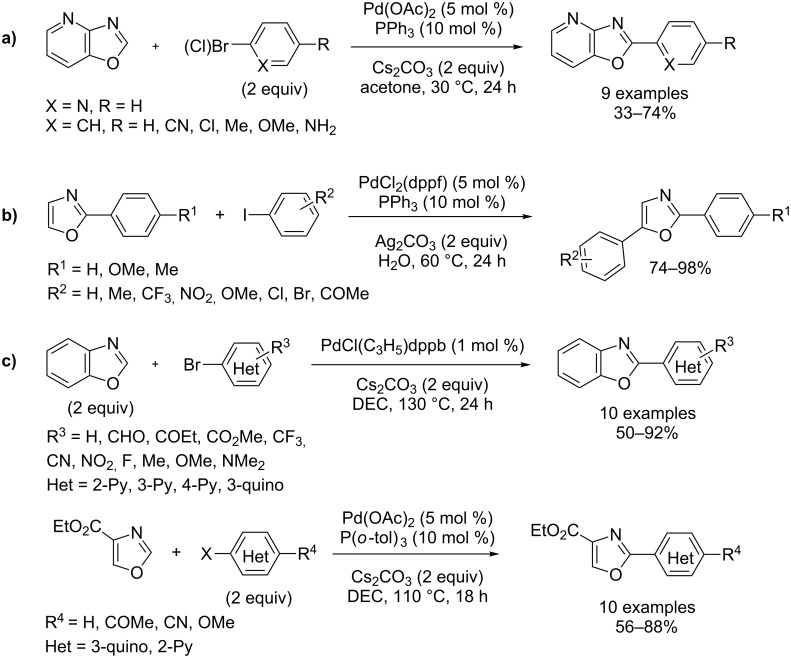
Palladium-catalyzed direct arylation of oxazoles under green conditions; (a) Zhuralev direct arylation of 2-azabenzoxazole at low temperature [57–58]; (b) Greaney direct arylation of oxazole in water [59–60]; (c) Hoarau–Doucet direct arylation of (benz)oxazole in diethylcarbonate [61–62].

More user-friendly conditions for palladium catalysis were also developed for the direct arylation of azoles by Zhuralev [57–58], Greaney [59–60], and Hoarau–Doucet [61–62] using, respectively, acetone solvent at low temperature, water as dispersing agent and diethylcarbonate (DEC) (Scheme 14).

**Scheme 15:**
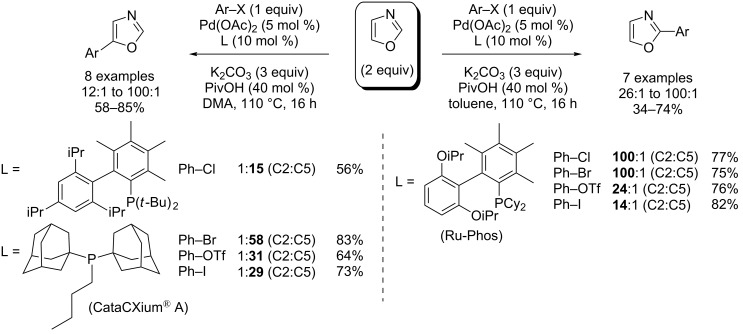
Pd(0)-catalyzed C2- and C5-selective (hetero)arylation of oxazole [63].

In 2010, Strotman and Chobanian reported the first highly challenging C2- and C5-selective Pd(0)-catalyzed direct arylation of oxazole with arylbromides, chlorides and triflates (Scheme 15 ) [63]. Interestingly, under the same catalytic conditions, the C2 (versus C5) position was preferred in nonpolar toluene solvent (versus polar DMF solvent) with RuPhos ligand (versus CataCXium^®^ A or 3,4,5,6-tetramethyl-*tert*-butyl-XPhos ligands).

**Scheme 16:**
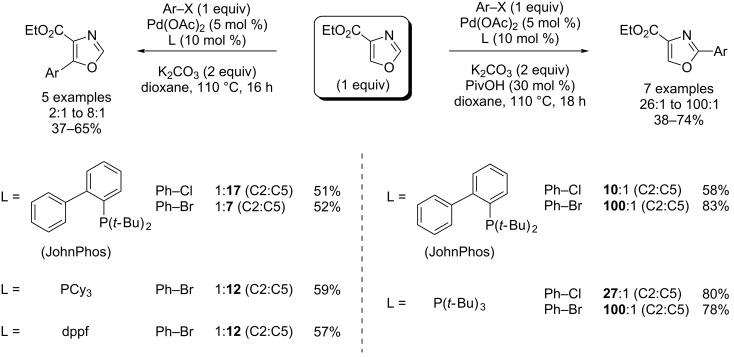
Pd(0)-catalyzed C2- and C5-selective (hetero)arylation of ethyl oxazole-4-carboxylate [64].

This year, Hoarau proposed the C2- and C5-regioselective Pd(0)-catalyzed direct (hetero)arylation of ethyl oxazole-4-carboxylate with arylbromides and chlorides in dioxane solvent by using K_2_CO_3_ as base. The C2 position was attained by using specifically P(*t*-Bu)_3_/PivOH or JohnPhos/PivOH pairs, whilst PCy_3_/PivOH pair or PCy_3_, JohnPhos or dppf ligands used alone allowed reversing of the selectivity in favour of the C5 position (Scheme 16) [64].

**Scheme 17:**
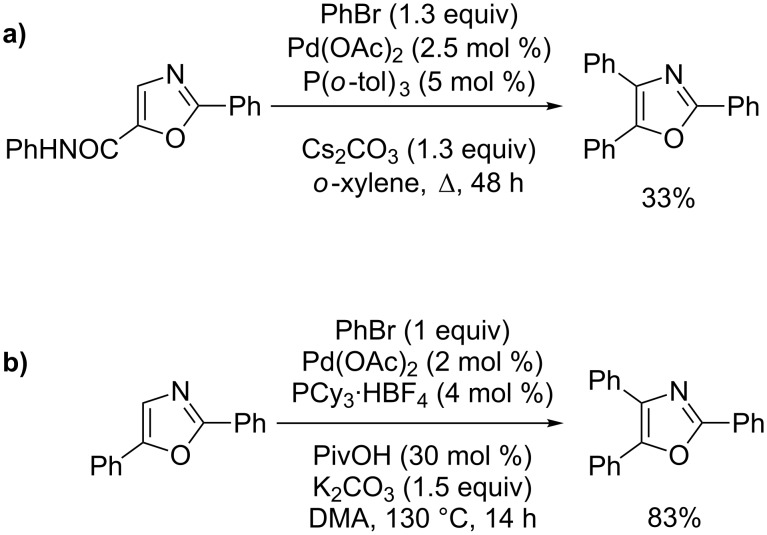
Pd(0)-catalyzed direct C4-phenylation of oxazoles; (a) Miura’s procedure [65]; (b) Fagnou’s procedure [66].

Miura first highlighted the reactivity of the C4 position of the oxazole ring in a direct substitutive-coupling methodology by reacting *N*-phenyl-2-phenyloxazole-5-carboxamide with phenylbromide (Scheme 17) [65]. Nevertheless, the introduction of a phenyl group also occurred subsequently at the C5 position, exclusively producing the 2,4,5-triphenyloxazole. Fagnou then reported the direct C4-phenylation of 2,5-diphenyloxazole with phenylbromide by using a general catalysis that had proved to be useful in catalytic direct arylation of azoles (Scheme 17) [66].

#### Palladium- and copper-catalyzed direct (hetero)arylation with halides: Progress in mechanisms

**Scheme 18:**
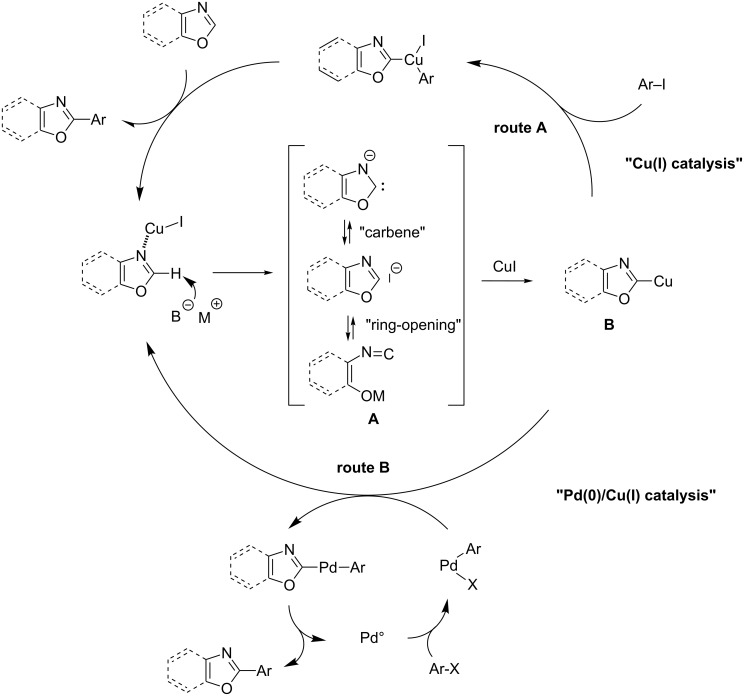
Catalytic cycles for Cu(I)-catalyzed (routeA) and Pd(0)/Cu(I)-catalyzed (route B) direct arylation of oxazoles with halides proposed by Bellina and Rossi [67].

Regarding the C5>C4>C2-reactivity scale of electron-rich azoles under electrophilic reaction, Miura proposed an SEAr-type mechanism for selective C5-arylation of imidazoles, thiazoles and oxazoles. [40] However, the C2-selectivity observed in the presence of Cu(I) salts as cocatalyst, or used alone, was suspected to arise from a proton–metal exchange of the most acidic position leading to an organocopper intermediate suitable for a nucleophilic substitution reaction. Daugulis reported a first rationalized route for the direct arylation of azoles, including oxazoles, by using a strong base under Cu(I) catalysis and based upon the previous formation of the oxazol-2-ylcuprate intermediate suitable in a subsequent oxidative step with aryliodide (Scheme 18, route A) [45]. However, Bellina and Rossi underlined the fact that the initial C2-oxazolylcopper formation stays currently unclear. Thus, they suggested a copper-induced reinforcing-acidity effect to facilitate the C2-deprotonation step, which could then be ensured by a very weak base-like caesium fluoride or even by DMF solvent (Scheme 18) [67]. The resulting C2-carbanion may be in equilibrium with a stabilized carbene intermediate and finally reacts with copper iodide to give the organocopper intermediate. Bellina and Rossi also noted that this last transmetalation step may be complicated by a second, well-known equilibrium of the 2-metallated oxazole with its ring-open tautomer [67]. Using Pd(0)/Cu(I) catalysis, the C2-cuprated oxazole may act as a transmetallating agent through a standard cross-coupling reaction (Scheme 18, route B) [67].

**Scheme 19:**
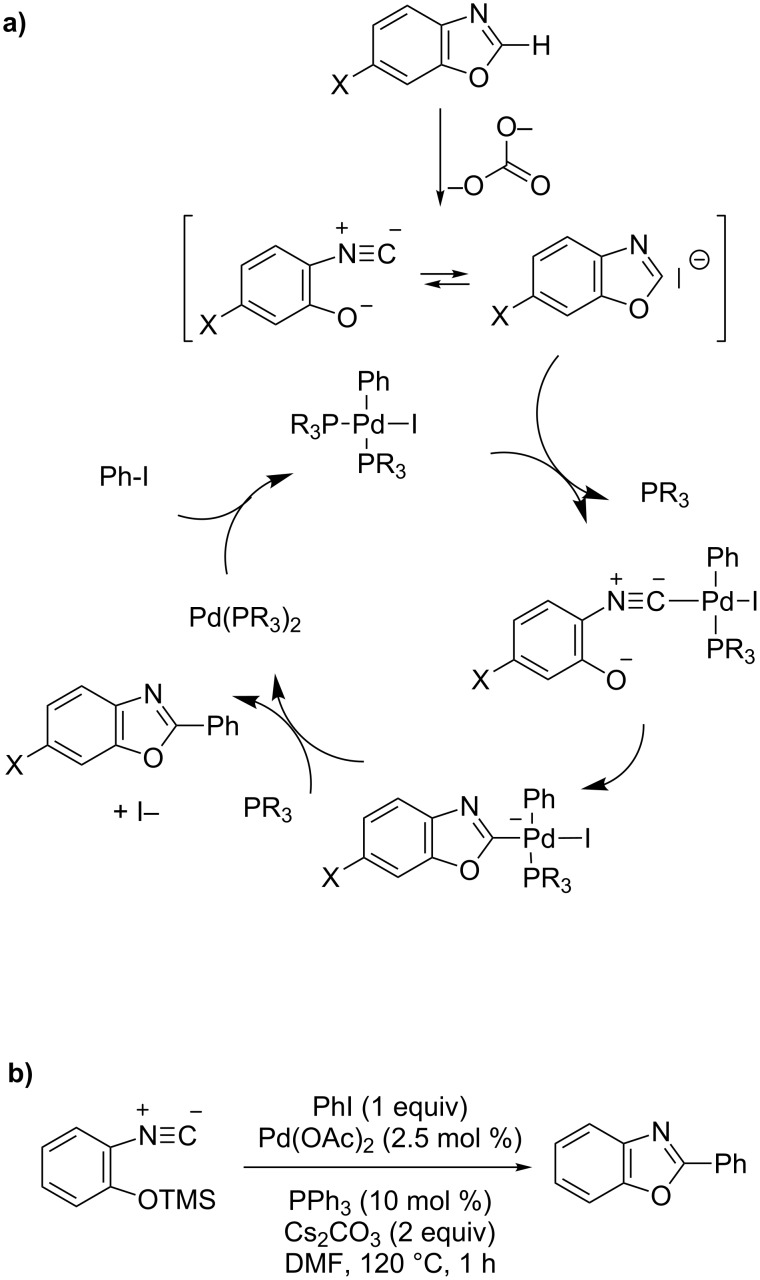
Base-assisted, Pd(0)-catalyzed, C2-selective, direct arylation of benzoxazole proposed by Zhuralev [58]; (a) Proposed cross-coupling-type mechanism; (b) Ring-close direct C2-arylation.

Under Pd(0)- and Cu(I)-free catalysis, Zhuralev identified a cross-coupling-type mechanism for direct arylation of benzoxazole and the aza-analogues using a strong base [57–58]. Thus, the arylpalladium complex is engaged in a Passerini-type reaction with the 2-metallated benzoxazole ring-open tautomer, leading directly to the ring-close benzoxazol-2-yl(aryl)palladium complex, and finally to the 2-arylated benzoxazole after a reductive elimination step (Scheme 19a). Deuterium-incorporation experiments and DFT calculations highly support this pathway as well as the successful palladium-catalyzed arylation of the *O*-silylated 2-isonitrilephenolate (Scheme 19b). Last year, Strotman and Chobanian retained this cross-coupling-type mechanism for their recently developed protocol for the Pd(0)-catalyzed, highly C2-selective, direct (hetero)arylation of oxazole [63].

**Scheme 20:**
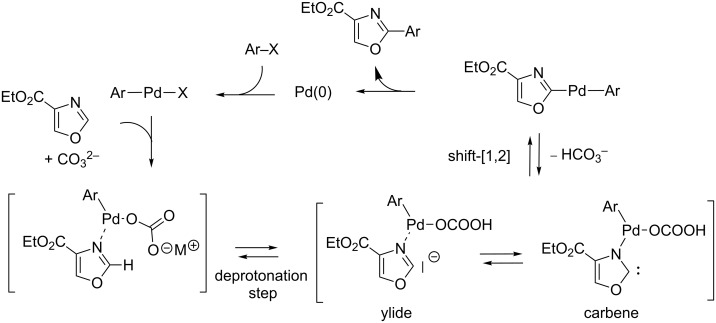
Electrophilic substitution-type mechanism proposed by Hoarau [64].

By contrast, Hoarau recently discarded the cross-coupling-type mechanism in favour of the direct (hetero)arylation of the more acidic oxazole-4-carboxylate employing strong Cs_2_CO_3_, K_3_PO_4_ or DBU bases, through deuterium-incorporation experiments in dioxane and toluene solvents, which led in both cases to the production of C2 and C5 deuterated ethyl oxazole-4-carboxylate [64]. Thus, an electrophilic substitution-type mechanism is preferred which is more in accordance with previous observations and a specific directed nitrogen-chelating effect (Scheme 20).

Thus, C2-selectivity may arise from a prior interaction of the palladium catalyst with nitrogen. The coordination of oxazole to arylpalladium(II) complex may lower the p*K*_a_ of oxazole more significantly and promote the deprotonation, and, as well, it may prevent the formation of the ring-open tautomer, according to Vedej’s observations of the high stability of the 2-lithiated oxazole previously coordinated with triethylborate [68].

**Scheme 21:**
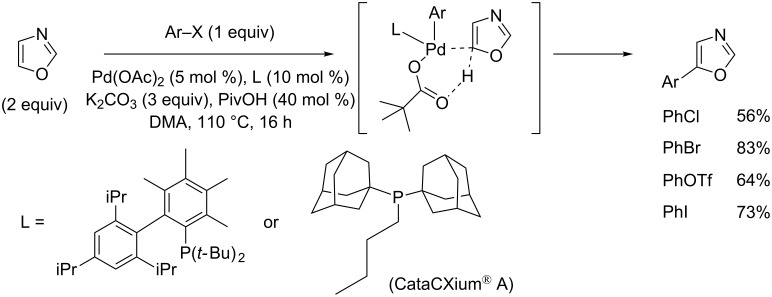
CMD-proceeding C5-selective direct arylation of oxazole proposed by Strotman and Chobabian [63].

In parallel to the emergence of a strong-base-assisted, nonconcerted, metalation–deprotonation mechanism evoked for the C2-selective direct substitutive coupling of electron-rich azoles under strong base conditions, the last five years has witnessed the particular ascension of the Pd(0)-catalyzed carbonate or pivalate-assisted concerted metalation–deprotonation (CMD) mechanism [19,69], which has now proved to be effective for a broad range of aromatics and heteroaromatics, including electron-rich as well as electron-deficient heterocycles [20–21,70]. Although Fagnou demonstrated the good reactivity of several azoles under CMD conditions [70], Strotman and Chobanian were the first to favour a CMD mechanism for their methodology for the highly C5-selective direct arylation of oxazole based upon the use of potassium carbonate and pivalate bases (Scheme 21) [63]. As the main argument, a strong pivalate-assisted effect was observed.

**Scheme 22:**
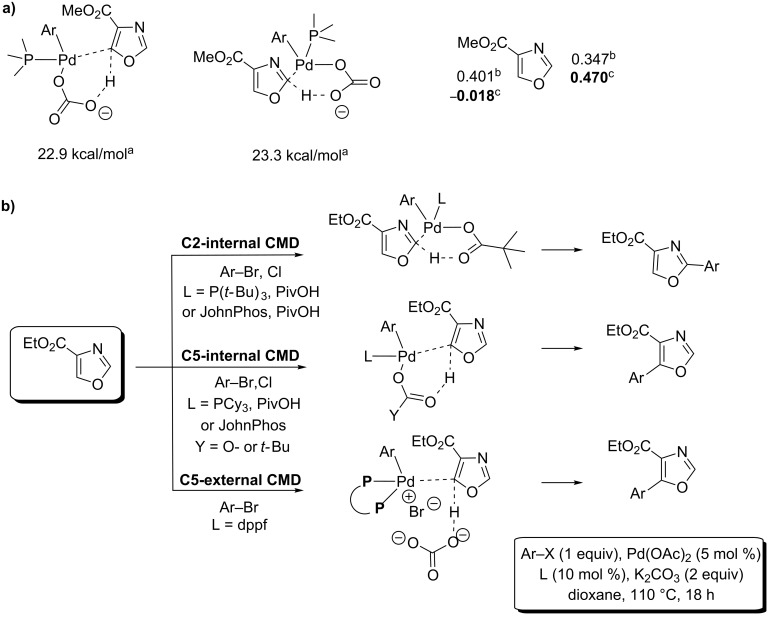
DFT calculations on methyl oxazole-4-carboxylate and consequently developed methodologies for the Pd(0)-catalyzed C2- and C5-selective direct arylations proceeding by CMD [64]; (a) DFT calculations on oxazole-4-carboxylate; (b) Developed novel CMD direct arylation methodologies. ^a^TS CMD Free Gibbs energy; ^b^HOMO coefficient; ^c^partial charge (ESP).

This year, after demonstrating that the C5 (versus C2) position is slightly favoured under a carbonate-assisted internal CMD mechanism in the oxazole-4-carboxylate series, through DFT calculations of the Gibbs free energy of the CMD transition state calculated at the C2 and C5 positions (Scheme 22a), Hoarau developed novel methodologies for the C2- and C5-selective direct arylation proceeding by CMD and using aryl bromides and chlorides (Scheme 22b) [64]. In particular, the charge-control interaction was identified as the main discriminating parameter since the HOMO levels are identical at both C2 and C5 positions (Scheme 22a). Thus, C2 selectivity was only attained by using the highly steric P(*t*-Bu)_3_/PivOH pair, whereas the less electron-donating tri(alkyl)arylphosphines as well as bidentate ligands were prompted to form an aryl-palladium complex possessing a highly electrophilic character, leading to C5-arylation.

#### Catalytic direct arylation of (benz)oxazoles with (pseudo)halides, carboxyarenes and organometallics

**Scheme 23:**
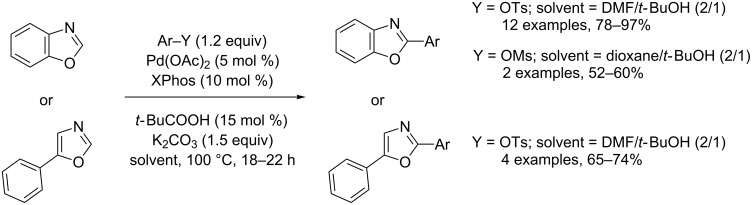
Pd(0)-catalyzed direct arylation of (benz)oxazoles with tosylates and mesylates [71].

Ackermann was the first to find convenient conditions for the base-assisted, Pd(0)-catalyzed direct substitutive coupling of heteroarene applicable to tosylate and mesylate electrophiles, which are prepared from inexpensive and easily available phenol derivatives (Scheme 23) [71].

**Scheme 24:**
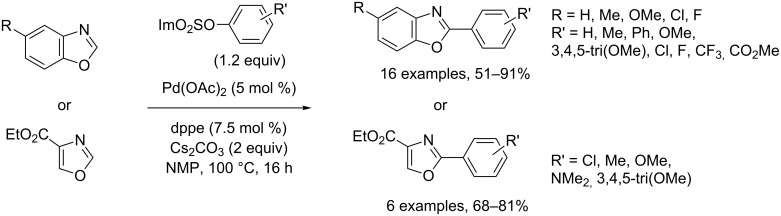
Pd(0)-catalyzed direct arylation of oxazoles with sulfamates [72].

More user-friendly sulfamates also proved to be convenient arylating agents in Pd(0)-catalyzed direct substitutive arylation of various oxazole series (Scheme 24) [72].

**Scheme 25:**
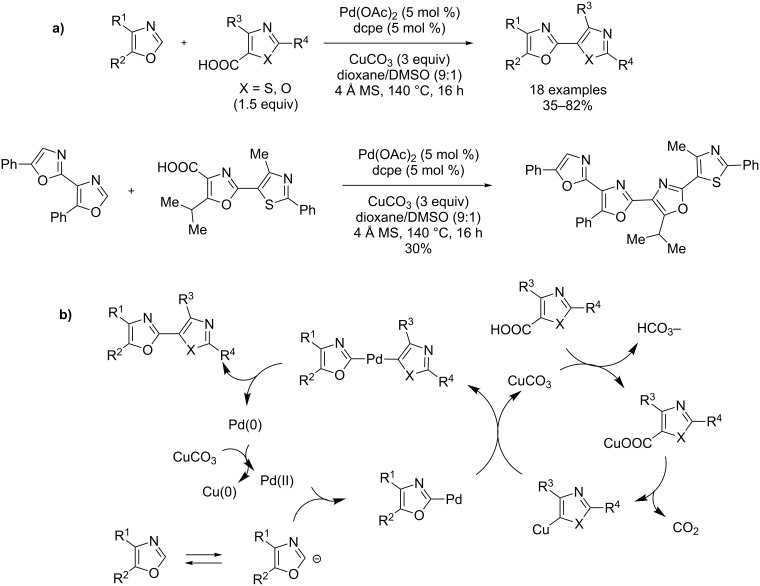
Pd(II)- and Cu(II)-catalyzed decarboxylative direct C–H coupling of oxazoles with 4- and 5-carboxyoxazoles [73]; (a) procedure; (b) proposed mechanism.

The first remarkable examples of catalytic, decarboxylative direct arylation of azoles were recently reported by the groups of Greaney and Tan [73–74]. Greaney selected the oxazole-4-carboxylate esters and 4-carboxyoxa(thia)zoles as substrates to prepare the naturally occurring 2,4-linked bis(azole). Interestingly, poly(azole) structures are also prepared by repeating the decarboxylative direct C–H cross-coupling sequence with the residual ester group (Scheme 25a) [73]. Mechanistically, a Cu(II)-catalyzed decarboxylation reaction produces the C4-cuprated azole, which intercepts the arylpalladium acetate complex produced by prior palladation of the substrate at the C2-position, to form the diazolylpalladium complex as the key intermediate leading finally to the bisazole system (Scheme 25b). Thus interestingly, the Cu(II) catalyst is used as a decarboxylating agent as well as for the reoxidation of Pd(0)-generated at the end of the catalytic cycle.

**Scheme 26:**
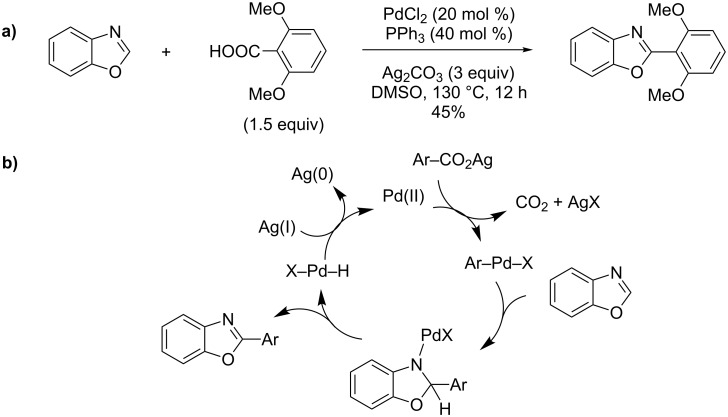
Pd(II)- and Ag(II)-catalyzed decarboxylative direct arylation of (benzo)oxazoles [74]; (a) procedure; (b) proposed mechanism.

Tan therefore used a copper-free, Pd(II)-based catalyst for direct decarboxylative cross-coupling of azole with various benzoic acids (Scheme 26a). In particular, benzoxazole was successfully coupled with 2,6-dimethoxybenzoic acid in 45% yield. Thus, without the assistance of a strong base, a carbopalladation was proposed as a key activation step of the benzoxazole by the arylpalladium complex, produced by a well-established silver-catalyzed decarboxylative palladation reaction. As its second role, the Ag(II) salt serves as a reoxidizing agent (Scheme 26b) [74].

**Scheme 27:**
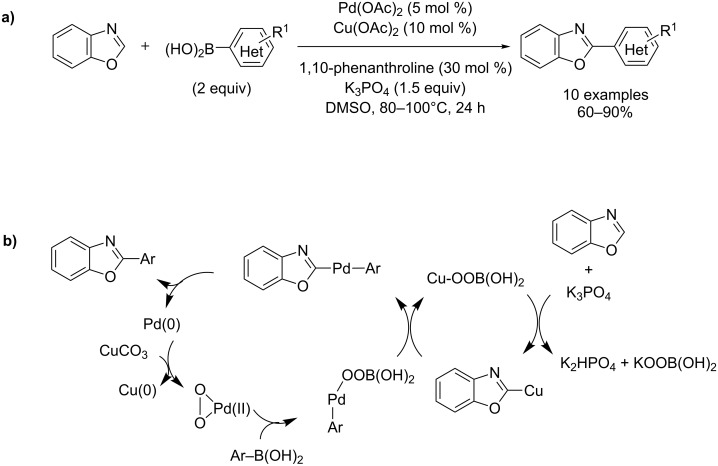
Pd(II)- and Cu(II)-catalyzed direct arylation of benzoxazole with arylboronic acids [76]; (a) procedure; (b) proposed mechanism.

**Scheme 28:**
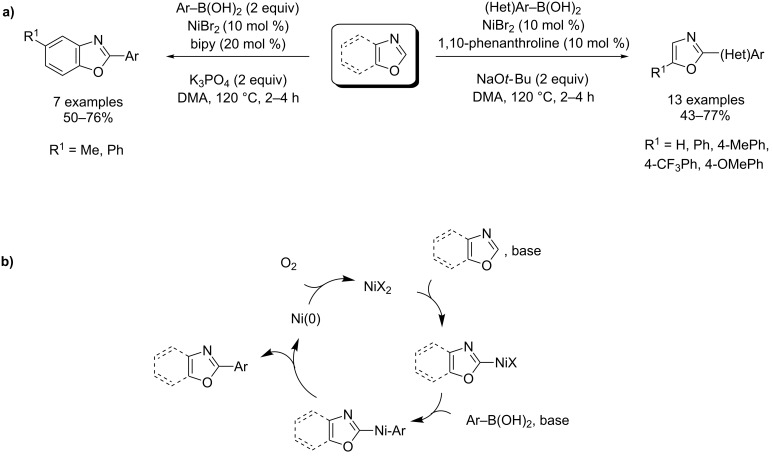
Ni(II)-catalyzed direct arylation of benzoxazoles with arylboronic acids under O_2_ [76]; (a) procedure; (b) proposed mechanism.

Recently, arylsilanes and arylboronic acids were also proposed as novel arylating agents in the strong-base-assisted, Pd(II)- and Ni(II)-catalyzed direct coupling of azole, by Liu, Hirano and Miura (Scheme 27a and Scheme 28a) [75–77].

Mechanistically, the Cu(II)-assisted palladation of the C2 position of benzoxazole is followed by a transmetalation step with arylboronic acids providing the arylazolylpalladium complex, which delivers the product (Scheme 27b and Scheme 28b).

#### Rhodium- and nickel-catalyzed direct arylation of oxazoles with halides

**Scheme 29:**
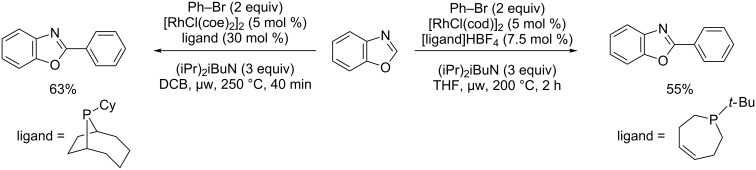
Rhodium-catalyzed direct arylation of benzoxazole [78–79].

The methodology for the Rh(I)-catalyzed direct substitutive coupling of azoles with halides was developed by the Bergman and Ellman group. In particular, the direct arylation of benzoxazole was performed under microwave activation with phenyl bromide (Scheme 29) [78–79].

**Scheme 30:**
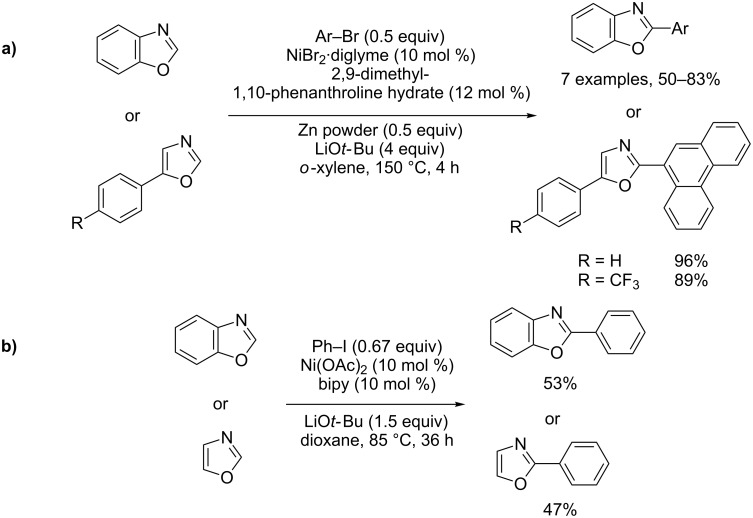
Ni(II)-catalyzed direct arylation of (benz)oxazoles with aryl halides; (a) Itami's procedure [80]; (b) Miura's procedure [81].

In 2009, Miura and Itami separately proposed the first convenient procedures for the direct substitutive coupling of azoles under Ni(II) catalysis with arylbromides (Scheme 30) [80–81].

#### Transition metal-catalyzed dehydrogenative cross-coupling

**Scheme 31:**
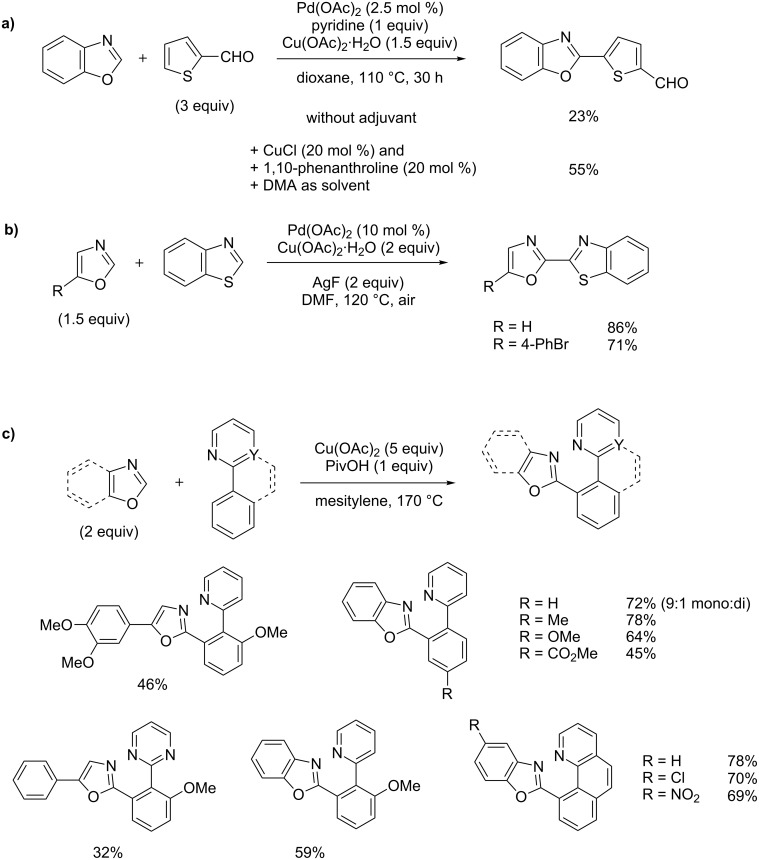
Dehydrogenative cross-coupling of (benz)oxazoles; (a) Pd(II)- and Cu(II)-catalyzed cross-coupling of benzoxazole with thiophene [82]; (b) Pd(II)- and Cu(II)-catalyzed cross-coupling of oxazoles with benzothiophene [83]; (c) Cu(II)-catalyzed direct cross-coupling of (benz)oxazole with arenes [84].

Last year, Hu and You reported the first extended study of Pd(II)- and Cu(II)- catalyzed oxidative C–H/C–H cross-coupling of electron-rich heteroarenes, including benzoxazole coupled with 2-formylthiophene by using Cu(I) cocatalyst and 1,10-phenanthroline in DMA solvent (Scheme 31a) [82]. This year, Oliaf studied more specifically the palladium- and copper-catalyzed oxidative C–H/C–H cross-coupling of various electron-rich 1,3-diazoles and reported notably the direct coupling of benzothiazole with two oxazoles, interestingly without a ligand but by using silverfluoride as cocatalyst (Scheme 31b) [83]. Miura recently reported the first remarkable palladium-free, Cu(II)-mediated direct oxidative C–H/C–H cross-coupling of arenes, using oxazoles and 2-arylazines as coupling partners (Scheme 31c) [84].

## Conclusion

The direct arylation of (hetero)arenes through the cleavage of C–H bonds has been proved to be a viable alternative to standard cross-coupling reactions. (Benz)oxazoles have drawn particular attention and have often been separately studied as a highly challenging and valuable heterocycle series. Since the pioneering works of Ohta and Miura in the 1990s, the direct C–H substitutive coupling of (benz)oxazoles has been intensively studied under Pd(0)- and/or Cu(I)- and, more recently, Rh(I)- and Ni(0)- catalysis by using aryl (pseudo)halides, including less-expensive aryl chlorides, tosylates, mesylates and phosphonates. Research efforts in this field are now focused on mechanism considerations since the broad diversity of catalytic metalation pathways represents undoubtedly an attractive tool for regioselectivity and the development of novel methodologies. Up until very recently, novel catalytic direct arylations of oxazoles have been developed. Arylboronic and carboxy(hetero)arene acids have thus been proposed as coupling partners under base- and copper-assisted Ni(II)- or Pd(II)-catalysis. Additionally, the first examples of highly attractive Cu(II)- or Pd(II)-catalyzed dehydrogenative couplings of (benz)oxazoles with (hetero)arenes have been developed.
